# A systematic review of inherited retinal dystrophies in Pakistan: updates from 1999 to April 2023

**DOI:** 10.1186/s12886-024-03319-7

**Published:** 2024-02-05

**Authors:** Asad Munir, Salma Afsar, Atta Ur Rehman

**Affiliations:** Department of Zoology, Faculty of Biological and Health Sciences, Hazara University, Mansehra, 21300 Khyber Pakhtunkhwa Pakistan

**Keywords:** Inherited retinal degenerations, Consanguinity, Homozygous, Monogenic disorders, Pakistan

## Abstract

**Background:**

Inherited retinal degenerations (IRDs) are a group of rare genetic conditions affecting retina of the eye that range in prevalence from 1 in 2000 to 1 in 4000 people globally. This review is based on a retrospective analysis of research articles reporting IRDs associated genetic findings in Pakistani families between 1999 and April 2023.

**Methods:**

Articles were retrieved through survey of online sources, notably, PubMed, Google Scholar, and Web of Science. Following a stringent selection criterion, a total of 126 research articles and conference abstracts were considered. All reported variants were cross-checked and validated for their correct genomic nomenclature using different online resources/databases, and their pathogenicity scores were explained as per ACMG guidelines.

**Results:**

A total of 277 unique sequence variants in 87 distinct genes, previously known to cause IRDs, were uncovered. In around 70% cases, parents of the index patient were consanguineously married, and approximately 88.81% of the detected variants were found in a homozygous state. Overall, more than 95% of the IRDs cases were recessively inherited. Missense variants were predominant (41.88%), followed by Indels/frameshift (26.35%), nonsense (19.13%), splice site (12.27%) and synonymous change (0.36%). Non-syndromic IRDs were significantly higher than syndromic IRDs (77.32% vs. 22.68%). Retinitis pigmentosa (RP) was the most frequently observed IRD followed by Leber’s congenital amaurosis (LCA). Altogether, mutations in *PDE6A* gene was the leading cause of IRDs in Pakistani families followed by mutations in *TULP1* gene.

**Conclusion:**

In summary, Pakistani families are notable in expressing recessively inherited monogenic disorders including IRDs likely due to the highest prevalence of consanguinity in the country that leads to expression of rare pathogenic variants in homozygous state.

**Supplementary Information:**

The online version contains supplementary material available at 10.1186/s12886-024-03319-7.

## Introduction

Inherited retinal dystrophies (IRDs) are a group of clinically and genetically diverse eye disorders ranging in prevalence from 1 in 2000 to 1 in 4000 people globally [1, 2]. IRDs are broadly divided into two categories (i) non-syndromic, and (ii) syndromic types depending upon absence or presence of extra-ocular manifestations, respectively. Major clinical symptoms of non-syndromic IRDs include, but not limited to, night blindness or nyctalopia, color vision deficiency, photophobia, nystagmus, reduced visual acuity, day vision loss as well as central or peripheral vision loss. Syndromic IRDs, on the other hand, are known for systemic findings such as obesity, polydactyly, renal abnormalities, deafness, speech, and intellectual disability together with ocular symptoms [3–6]. Globally, mutations in over 300 distinct genes have thus far been associated with all forms of IRDs [3]. IRDs follow all modes of Mendelian inheritance such as autosomal recessive [7], autosomal dominant [8], X-linked [9], mitochondrial [10] as well as digenic [11] and oligogenic patterns [12]. Accordingly, IRDs are classified into various subtypes depending upon disease onset, mode of inheritance, rate of disease progression, clinical presentation, part of retina affected (rods, cones, retinal pigment epithelium, inner retina and choroid), and/or involvement of extra ocular phenotypes [13]. Non-syndromic and syndromic IRDs are briefly explained in the following sections.

### Non-syndromic IRDs

#### Cone or cone-rod dystrophy (CD/CRD)

Cone and cone-rod dystrophies (CD/CRD) are a rare form of retinal dystrophies with a worldwide prevalence rate of 1:40,000 [14]. CD/CRD are progressive disorders of cone and rod photoreceptor cells in retina presenting clinical and genetic heterogeneity [4]. Major clinical symptoms of CD include reduced day vision, color vision deficiency, reduced visual acuity, and photophobia [15]. Similarly, CRD is characterized by cone dysfunction at first resulting in progressive loss of day vision. This is followed by rods dysfunction eventually leading to night blindness (nyctalopia). However, symptoms such as photophobia, color vision deficiency, and legal blindness overlap between CD and CRD. The electroretinogram (ERG) shows both cone and rod dysfunction and is non-recordable in advanced case [16]. CD/CRD may transmit as autosomal recessive, autosomal dominant or X-linked entity with mutations in as many as 32 distinct genes identified so far [17]. Gill and colleagues have reported that 62.2% cases of recessively inherited CD/CRD are linked to mutations in *ABCA4* gene. Similarly, of the dominantly inherited CD/CRD, 34.6% cases are attributed to mutations in *GUCY2D* gene. Lastly, of the X-linked inherited CD/CRD, 73.0% cases are due to mutations in *RPGR* gene [18].

### Congenital stationary night blindness (CSNB)

As the name indicate, congenital stationary night blindness (CSNB) is a form of non-progressive inherited retinal dystrophy that appears at birth, however identified in childhood [19]. Visual symptoms include night blindness, nystagmus and reduced visual acuity [20]. ERG findings show normal cone response; however, reduced or abolished rod response is detected on ERG in CSNB. Similarly, fundoscopy mostly remains unremarkable [21]. CSNB is divided into four distinct types namely, Schubert–Borstein type, Riggs type, Oguchi disease, and fundus albipunctatus [22]. Though patients with Schubert–Borstein or Riggs type both have normal fundi, the two types can be distinguished from each other with the help of full-field electroretinography (ff-ERG) [23]. Oguchi disease and fundus albipunctatus shows fundus abnormalities. For instance, Oguchi disease is characterized by a gray-white metallic sheen that disappears after dark adaptation, a feature called the Mizuo–Nakamura phenomenon. Fundus albipunctatus is characterized by small white dots scattered across the posterior pole sparing the fovea. In scotopic settings, Riggs type has flat a-wave in dim flash and reduced a- and b-wave with a strong single flash. In contrast, the Schubert–Bornschein type has normal a-wave and severely reduced b-wave, classically described as an electronegative waveform. In photopic settings, Riggs type has a normal photopic response, the Schubert–Bornschein type has abnormal photopic findings [23–25]. To our knowledge, at least 18 genes are known to cause CSNB, including 13 genes for AR-CSNB, 3 genes for AD-CSNB, and 2 genes for XL-CSNB [20].

### Leber congenital amaurosis (LCA)

Leber’s congenital amaurosis (LCA) is a rare, and one of the most clinically severe form of IRDs with a worldwide prevalence of 1:80,000 [26]. LCA is, typically, associated with early onset vision loss, nystagmus, and amaurotic pupils [27]. Clinical pattern includes pigmentory retinopathy, reduced or absent ERG response, poor central vision or complete blindness at birth [28]. LCA predominately follows autosomal recessive inheritance pattern except for *CRX*, *IMPDH1*, and *OTX2* genes that results in autosomal dominant LCA [29]. LCA account for approximately 5% of the total IRDs cases [30], and ~ 20% of childhood blindness [27]. So far, pathogenic variants in 38 genes are known to cause LCA [27]. Some of the frequently mutated genes in LCA include *AIPL1*, *CEP290*, *CRB1* and *GUCY2D* [31].

### Macular degeneration (MD)

Macular degeneration (MD) is a heterogeneous group of progressive eye disorders that are clinically characterized by bilateral symmetrical macular abnormalities and macular flecks. Visual complaints include, reduced visual acuity, central vision loss, photophobia, slow dark adaptation, and nystagmus [32]. Stargardt disease 1 (STGD1) disease is the most common form of macular dystrophy with a prevalence rate of 1 in 8000–10000 [33]. STGD1 follows an autosomal recessive mode of inheritance with *ABCA4* as the leading cause of the disease that accounts for 95% STGD1 cases [34]. Of the total 18 genes known to cause MD, 14 genes are responsible for AD-MD while the remaining 4 genes results in AR-MD [RetNet—Retinal Information Network (uth.edu), accessed on March 6, 2023].

### Retinitis pigmentosa (RP)

Retinitis pigmentosa (RP), with a worldwide incidence rate of 1:3000 to 1:4000, appears as the most highly frequent type of IRDs [35]. Though symptoms and onset of RP is largely variable, it usually starts with night blindness during first or second decade of life due to the degeneration of rod photoreceptor cells. This is followed by visual field constriction (tunnel vision) and degeneration of cone photoreceptors, finally leading to complete blindness by late third or early fourth decade of life. Auxiliary symptoms may include photophobia, nystagmus and reduced visual acuity [35]. Around 70%-80% of RP cases are non-syndromic (isolated) while the remaining 20–30% cases are associated with non-ocular manifestations, and thus classified as syndromic RP [36]. RP may be inherited as an autosomal dominant (15–25%), autosomal recessive (5–20%), X-linked (5–15%) [37, 38], or a di-genic entity [39] with associated mutations reported in over 132 different genes [RetNet—Retinal Information Network (uth.edu)]. Mutations in *RPGR* gene is the leading cause of X-linked RP [40].

### Syndromic IRDs

#### Bardet-biedl syndrome

Bardet-Biedl syndrome (BBS) is a rare ciliopathy with multisystem involvement [41]. Hallmark features of BBS include obesity, macro or micro-cephaly, night blindness, cone-rod dystrophy, retinitis pigmentosa (RP), hypodontia, dental crowding, hepatic fibrosis, hypogonadism, hypogenitalism, tricuspid regurgitation, dilated cardiomyopathy, renal anomalies, polydactyly, brachydactyly, syndactyly, delayed development, mental retardation, ataxia, speech disability speech delay, and diabetes mellitus [42, 43]. However, expression of these symptoms may vary from one person to the other. Since the mutated genes in BBS have functional relevance in ciliary biogenesis and trafficking, the condition is regarded as ciliopathy [44]. As per literature survey [45], and RetNet—Retinal Information Network (uth.edu), mutations in 28 distinct genes/loci are so far associated with BBS phenotypes, all following an autosomal recessive inheritance pattern [46] and in some cases oligogenic [47]. Interestingly, ~ 50% of the total BBS cases are attributed to pathological sequence variations in three genes, namely, *BBS1*, *BBS2*, and *BBS10* [45].

### Usher syndrome (USH)

Usher syndrome (USH) is a rare form syndromic IRDs, presenting deafness in conjunction with blindness, that affect people with a worldwide prevalence of 4–17 in 100000 [48] [49]. Chief ocular complaints are night blindness, progressive vision loss, nystagmus whereas non-ocular symptoms include hearing difficulty or sensorineural hearing loss of variable degree [50]. USH is sub-divided into different types, for example, USH1, USH2 and USH3, based upon clinical presentation of the disease. USH2 is the predominant type among all sub-types [51]. USH is passed in an autosomal recessive pattern [52], and di-genic [53] with mutation in 10 genes thus far known to cause the disease. While mutations in *USH2A* gene account for roughly 80% of USH2 cases, *MYO7A* gene mutations are responsible for over 50% of USH1 cases [54].

### Joubert syndrome

Joubert syndrome (JBTS) is an infrequent genetic ciliopathy characterized by the involvement of multiple systems and organs, including the brain, kidneys, liver, and eyes. The clinical presentation of JBTS typically involve mild to moderate mental retardation, macrocephaly, retinal dystrophy, nystagmus, coloboma, visual impairment, hepatic and renal anomalies, and skeletal deformities with characteristic “molar tooth sign” on MRI. Given its rarity as a genetic ciliopathy, JBTS has a global occurrence rate of 1 in 80,000 to 1 in 100,000 live births [55]. JBTS is predominantly inherited as an autosomal recessive disorder [56]. However, there have been reports of X-linked JBTS due to mutations in the *OFD1* gene [55]. Pathogenic sequence variations in at least 40 genes have been reported to cause JBTS so far [57]. Mutations in *AIH1* and *CEP290* genes collectively account for approximately 38% of genetically diagnosed JBTS patients [55].

### Senior-loken syndrome

Senior-Loken syndrome (SLS) is a rare syndromic form of IRDs that is estimated to affect 1 in 1,000,000 individuals worldwide. The disease is characterized by retinopathy that may progress as Leber congenital amaurosis (LCA), retinitis pigmentosa (RP), or sector RP. Patients typically present with photophobia, nystagmus, and hyperopia, which may manifest in the first few years of life or later in childhood. Additionally, patients with SLS experience nephronophthisis, a condition that is marked by cystic kidney disease (medullary cystic kidney disease), reduced concentrating ability, and chronic tubule-interstitial nephritis. Over time, the disease typically progresses to end-stage renal disease [58, 59]. SLS is inherited as an autosomal recessive Mendelian disorder [60]. To date, mutations in 10 genes are associated with SLS including *NPHP1* [61], *NPHP2* [62], *NPHP3* [63]*, NPHP4* [64], *NPHP5/IQCB1* [65], *NPHP6/CEP290* [66, 67], *NPHP10/SDCCAG8* [68], *NPHP13/WRD19* [69], *NPHP15/CEP164* [70], and *TRAF3IP1* [71].

## Methods

This review was carried out between November-2022 and April-2023 by following PRISMA guidelines as mentioned in Supplementary Table S1. Briefly, the inclusion criteria for considering genetic studies comprised families of Pakistani descent, exhibiting both syndromic and non-syndromic inherited retinal disorders, and articles published during the period spanning from 1999 to April-2023. A thorough literature survey was performed for this purpose using PubMed (NCBI), Google Scholar, and Web of Science. Different key words such as inherited retinal degenerations/dystrophies, IRDs, retinitis pigmentosa (syndromic/non-syndromic), Stargardt disease, cone- and/or cone-rod dystrophy, macular degeneration, congenital stationary night blindness, leber congenital amaurosis, achromatopsia, color blindness, mutations, Pakistan, etc. were used for retrieving relevant literature. All articles that appeared during our search were further shortlisted and papers that did not fall under the purview of this review were excluded. This included articles presenting findings related to non-retinal phenotypes like cataract, age related macular dystrophy, microphthalmia, anophthalmia, aniridia, anterior segment dysgenesis, and sporadic cases. Data pertaining to genetic and clinical aspects of the families were extracted from all papers and recorded (and tabulated) in Microsoft (MS) excel sheet. This followed manual curation of the data using different options of the MS excel tool. Genomic nomenclature of all variants reported in this study were validated using VariantValidator [https://variantvalidator.org]. Additional features of the variants such as impact of the variations on cDNA and protein level, gene symbols, protein and transcript ID, genomic coordinates of the variation, inheritance pattern, and allele frequencies were determined using major genomic data bases (Ensemble, UCSC, HGNC, OMIM, gnomAD). All identified variants were queried in different online databases such as ClinVar, HGMD and Varsome to check their clinical significance. ACMG verdicts about the variants were retrieved from Varsome database. Retinal information network (RetNet) was used to calculate number of genes associated with each IRDs type. All missense variants were evaluated for their predicted pathogenicity using different *in-silico* tools, namely, CADD, DANN, LRT, Mutation Assessor, Mutation Taster, Mutpred, PolyPhen-2, PROVEAN, and SIFT.

## Results

### Demographic and clinical features of IRDs

**Fig. 1:**
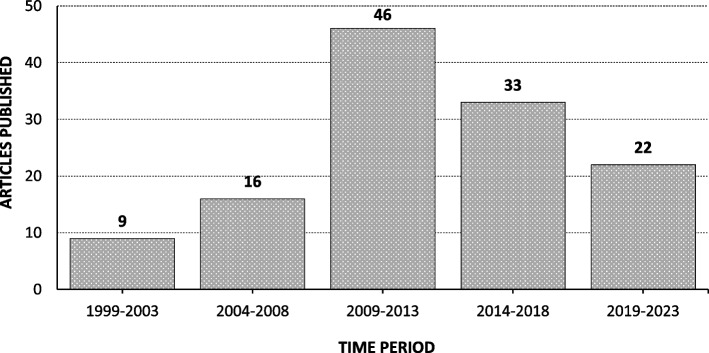
Chart showing number of articles published with respect to time (1999–2023)

This review paper focused on a total of 126 published research articles, including 1 case series, 34 case reports, and 91 cohort papers, all documenting IRDs in families of Pakistani descent between 1999 and April-2023. Excluding a retrospective case series study (*n* = 1), major diagnostic methods utilized in the remaining 125 articles included linkage analysis and/or homozygosity mapping coupled with targeted Sanger sequencing (*n* = 62), targeted Sanger sequencing (*n* = 24), and next-generation sequencing (panel-based sequencing, whole exome sequencing, and whole genome sequencing) (*n* = 39) (Fig. 1, Table S3).

All articles were thoroughly checked to infer different features such as ethnic affiliation of the patients, parental consanguinity, zygosity of the identified alleles, mutation types /molecular impact of the alleles, inheritance pattern, and disease type. Collectively, highest number of IRDs cases were reported from Punjab province (32.21%), followed by Khyber Pakhtunkhwa (13.73%), Sindh (1.12%), Baluchistan (0.84%), and Kashmir (< 1%), while a subset (1.12%) of studies documented IRDs in Pakistani families living abroad (UK/Canada). In the remaining 50.70% cases, no ethnic affiliation could be inferred from the published reports. Analysis of the retrieved articles for presence/absence of parental consanguinity revealed that parents were consanguineously married in 70.03% of the cases, 2.24% non-consanguineously married while no information about parental consanguinity were available in 27.73% cases. Interestingly, 95.50% IRDs cases were found to follow autosomal recessive (AR) inheritance pattern followed by autosomal dominant (AD) (1.70%), X-Linked (1.10%) pattern (Table 1). In the remaining 1.70% cases, information about inheritance pattern was not known. The authors, however, reported homozygous pathogenic variants in genes which can cause both AD and AR inheritance pattern (as per OMIM database).

**Table 1:** Demographic attributes of the index cases and genomic features of the observed alleles in Pakistani IRDs families between 1999 and April 2023

**Demographic characteristics (*n* = 357 index cases)**	**No.**	**%**
**Ethnicity**		
N.D	181	50.70
Punjab	115	32.21
Khyber Pakhtunkhwa	49	13.73
Britishi Pakistani	3	0.84
Sindh	4	1.12
Balochistan	3	0.84
Canadian Pakistani	1	0.28
Kashmir	1	0.28
**Parental consanguinity**		
Yes	250	70.03
N.D	99	27.73
No	8	2.24
**Clinical Diagnosis**		
Non-Syndromic	276	77.31
Syndromic	81	22.68
**Inheritance pattern**		
Autosomal Dominant	6	1.70
Autosomal Recessive	341	95.50
Autosomal Dominant/Recessive	6	1.70
X-Linked	4	1.10
**Genomic features of the observed alleles (*n* = 277 variants)**		
**Zygosity**		
Homozygous	246	88.81
N.D	22	7.94
Heterozygous	6	2.17
Hemizygous	3	1.08
**Impact Type**		
Missense	116	41.88
Indels/Frameshift	73	26.35
Nonsense	53	19.13
Splicing	34	12.27
Synonymous	1	0.36
**Classification of the observed missense variants (*n* = 116)**		
**ACMG**		
Benign/Likely Benign	8	6.90
Pathogenic/Likely Pathogenic	63	54.31
Uncertain Significance	45	38.79
**ClinVar**		
Benign/Likely Benign	6	5.17
Pathogenic/Likely Pathogenic	49	42.24
Uncertain Significance	16	13.79
Conflicting	9	7.76
Not reported	36	31.03
**Types of single nucleotide variants (SNVs) reported (*n* = 170)**		
Synonymous	1	0.59
Missense	116	68.24
Nonsense	53	31.18
**Transitions (*n* = 109)**		
Purine-Purine	45	41.28
Pyramidine-Pyramidine	64	58.72
**Transversions (*n* = 61)**		
Purine-Pyramidine	37	60.66
Pyramidine-Purine	24	39.34
***Transitions (n* = *109)***		
C > T	48	44.04
G > A	34	31.19
A > G	11	10.09
T > C	16	14.68
***Transversions (n* = *61)***		
G > T	17	27.87
C > A	10	16.39
G > C	12	19.67
T > G	9	14.75
A > T	6	9.84
C > G	3	4.92
T > A	2	3.28
A > C	2	3.28

**Fig. 2:**
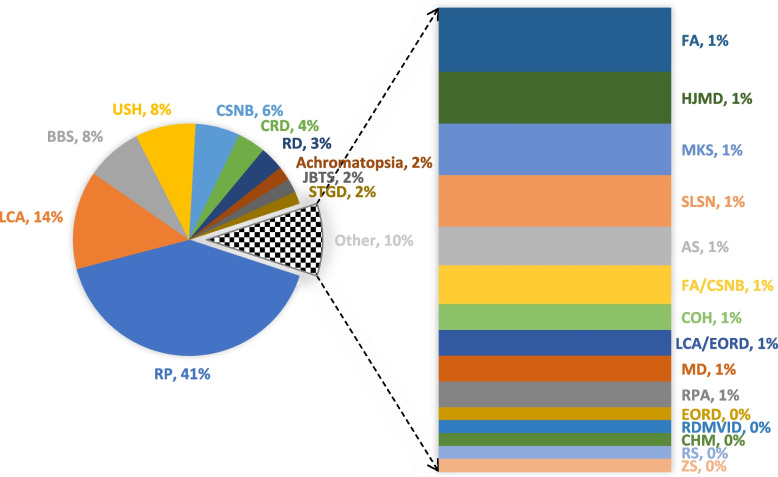
Pie chart showing frequency distribution of IRDs types reported in Pakistani families

As per available literature and genotype information, higher cases of non-syndromic (77.32%) IRDs were reported compared to syndromic IRDs (22.68%) (Table 1). Syndromic IRDs included Alström syndrome (AS) (~ 1%), BBS (8%), Cohen syndrome (COH) (~ 1%), JBTS (2%), MKS (1%), Retinal dystrophy with microvillus inclusion disease (RDMVID) (< 1%), SLSN (1%), USH (8%), Zellweger syndrome (ZS) (< 1%). Non-syndromic IRDs included RP (41%), LCA (14%), CSNB (6%), CRD (4%), RD (3%), Achromatopsia (2%), STGD (2%), Fundus Albipunctatus (FA) (1%), Hypotrichosis juvenile macular dystrophy HJMD (1%), FA/CSNB (~ 1%), LCA/EORD (~ 1%), MD (~ 1%), Retinitis punctata albescens (RPA) (~ 1%), Early onset retinal dystrophy (EORD) (< 1%), Choroideremia (CHM) (< 1%), Retinoschisis (RS) (< 1%) (Fig. 2).

### Genetic spectrum of IRDs

**Fig. 3:**
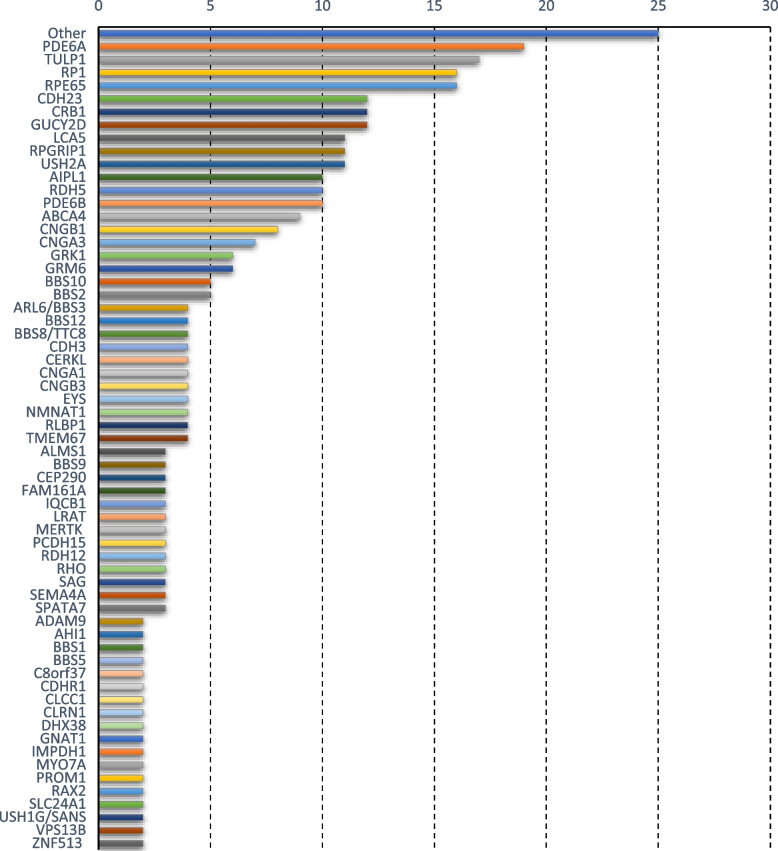
Bar chart showing frequently mutated IRDs-associated genes in Pakistani families

Various characteristics of the identified genomic variants are shown in Table 1. A total of 277 disease-causing alleles across 87 known IRDs-associated genes were documented in the literature under the scope of this study as mentioned in supplementary Table S2. Topmost mutated genes included *PDE6A* (5.32%), *TULP1* (4.76%), *RP1* (4.48%), *RPE65* (4.48%), *CDH23* (3.36%), *CRB1* (3.36%), *GUCY2D* (3.36%), *LCA5* (3.08%), *RPGRIP1* (3.08%), *USH2A* (3.08%), *AIPL1* (2.80%), *RDH5* (2.80%) *PDE6B* (2.80%) (Fig. 3). On the contrary, least frequently mutated genes, categorized as “Others”, were *ARL13B, ARL3, ASRGL1, BEST1*, *CC2D2A, CHM, DRAM2, IFT43, IMPG2, MKKS, MKS1, NPHP4, NR2E3, NYX, PDE6H, PEX6, PRCD, PRPF3, RBP3, RPGR, RS1, SLC6A6, SNRNP200, STX3* and *TCTN2* (each reported only once in Pakistani families so far) (Fig. 3). Of the total 277 disease-causing alleles found in Pakistani IRDs patients, 88.81% alleles were found in a homozygous state, 2.17% in heterozygous state, and 1.08% in hemizygous state; however, zygosity of the remaining 7.94% alleles was not described in the published reports (Table 1). Based on their predicted impact on protein, alleles were classified as missense, indels/frameshift, nonsense, splicing, and synonymous with their respective frequencies of 41.88%, 26.35%, 19.13%, 12.27%, and 0.36% (Table 1). Of the total 277 alleles, 61.37% (170 alleles) were defined as single nucleotide variants (SNVs). Of the 170 SNVs, 109 or 64.12% were transitions (Purine to Purine = 45, Pyramidine to Pyramidine = 64) and 61 or 35.88% were transversions (Purine to Pyramidine = 37, Pyramidine to Purine = 24) (Table 1). A C > T was the most frequent (44.04%) transition followed by G > A (31.19%), T > C (14.68%), and A > G (10.09%). Similarly, G > T (27.87%) was the mostly frequent transversion followed by G > C (19.67%), C > A (16.39%), T > G (14.75%), A > T (9.84%), C > G (4.92%), T > A (3.28%) and A > C (3.28%) (Table 1). Using ACMG guidelines, all reported alleles were re-evaluated for their clinical significance. Collectively, ‘pathogenic/likely pathogenic’ variants were found to be 71.48% followed by ‘variants of uncertain significance (VUS)’ (19.49%), and ‘benign/likely benign’ (2.89%). No ACMG verdict about the 6.14% alleles was available on the Varsome database (Supplementary Table S2).

Furthermore, all missense variants (*n* = 116) were re-evaluated as per ACMG guidelines/standards, ClinVar database, and using in-silico tools (Table 2). ACMG predicted 63 missense alleles as pathogenic/likely pathogenic, 45 as VUS, and 8 as benign/likely benign (Table 2). Similarly, ClinVar database showed 49 missense variants as pathogenic/likely pathogenic, 16 as VUS, 9 as conflicting, and 6 as benign/likely benign. Thirty-six missense alleles were; however, not reported in the ClinVar database. Findings of our in-silico study are shown in Table 2.

**Table 2:** In-silico predictions of the identified 116 missense variants reported in Pakistani families

**Gene**	**Transcript ID**	**cDNA**	**CADD**	**DANN**	**LRT**	**MA**	**MT**	**MP**	**PP-2**	**PRO**	**SIFT**	**ACMG**	**ClinVar**
AIPL1	NM_014336.5	c.465G > T	35	VUS	B	B	VUS	VUS	PD	VUS	VUS	VUS	VUS
SLC6A6	NM_003043.6	c.1196G > T	33	VUS	P	P	VUS	P	PD	n.a	n.a	LP	LP
RLBP1	NM_000326.5	c.346G > C	33	VUS	P	B	VUS	VUS	PD	B	B	P	LP
USH2A	NM_206933.4	c.7334C > T	32	VUS	B	VUS	B	VUS	PD	VUS	VUS	B	B
TULP1	NM_003322.6	c.1561C > T	32	VUS	P	P	VUS	P	PD	P	P	VUS	LP
ADAM9	NM_003816.3	c.1144 T > G	32	VUS	P	P	VUS	P	PD	P	P	VUS	n.a
SNRNP200	NM_014014.5	c.3269G > A	32	P	P	P	VUS	P	PD	VUS	P	LP	P
GUCY2D	NM_000180.4	c.2302C > T	32	P	VUS	P	B	n.a	PD	P	P	P	P
GNAT1	NM_144499.3	c.386A > G	32	VUS	P	P	VUS	P	PD	P	P	LP	P
ARL3	NM_004311.4	c.296G > T	32	VUS	P	P	VUS	P	PD	P	P	LP	P
PDE6A	NM_000440.3	c.1630C > T	32	P	VUS	VUS	B	n.a	PD	P	P	P	P/LP
RPE65	NM_000329.3	c.751G > T	32	VUS	P	P	VUS	P	PD	VUS	P	LP	VUS
PEX6	NM_000287.4	c.2626C > T	32	P	P	P	VUS	n.a	PD	P	VUS	LP	VUS
LRAT	NM_004744.5	c.196G > C	31	VUS	P	P	VUS	P	PD	P	P	LP	n.a
GUCY2D	NM_000180.4	c.2384G > T	31	VUS	VUS	P	B	P	PD	P	P	P	n.a
ARL13B	NM_001174150.2	c.236G > A	31	P	VUS	VUS	VUS	P	PD	VUS	P	LP	P
TULP1	NM_003322.6	c.1445G > A	31	P	P	P	VUS	n.a	PD	VUS	P	P	P
RPE65	NM_000329.3	c.131G > A	31	P	VUS	P	VUS	P	PD	VUS	P	P	P
RDH12	NM_152443.3	c.506G > A	31	P	P	VUS	VUS	P	PD	VUS	P	P	P/LP
GUCY2D	NM_000180.4	c.2384G > A	31	P	VUS	P	B	n.a	PD	VUS	P	LP	VUS
GUCY2D	NM_000180.4	c.582G > C	29.8	VUS	B	VUS	VUS	P	PD	P	P	VUS	n.a
RAX2	NM_001319074.4	c.236G > A	29.7	VUS	B	P	VUS	P	n.a	n.a	n.a	VUS	VUS
USH2A	NM_206933.4	c.12523 T > G	29.5	VUS	B	P	VUS	P	PD	P	P	P	n.a
PRPF3	NM_004698.4	c.1481C > T	29.4	VUS	P	P	VUS	P	PD	P	P	P	CFL
CNGB1	NM_001297.5	c.2284C > T	29.4	P	P	P	VUS	n.a	PD	P	P	P	P
PDE6B	NM_000283.4	c.1655G > A	29.3	P	P	P	VUS	P	PD	VUS	P	P	P/LP
DHX38	NM_014003.4	c.971G > A	29.1	P	VUS	P	VUS	B	PD	VUS	VUS	VUS	LP
CDH23	NM_022124.6	c.8150A > G	29	VUS	P	P	VUS	P	PD	P	P	VUS	n.a
RDH5	NM_002905.5	c.319G > C	28.8	P	P	VUS	VUS	P	PD	P	P	LP	n.a
USH1G/SANS	NM_173477.5	c.1373A > T	28.7	VUS	P	P	VUS	P	PD	n.a	n.a	P	P/LP
RPE65	NM_000329.3	c.179 T > C	28.6	VUS	P	VUS	VUS	P	PD	VUS	P	LP	n.a
RPE65	NM_000329.3	c.782 T > C	28.6	VUS	P	P	VUS	P	PD	P	P	LP	VUS
ABCA4	NM_000350.3	c.5882G > A	28.4	VUS	P	B	VUS	n.a	PD	P	P	P	P
TULP1	NM_003322.6	c.1466A > G	28.2	VUS	P	P	VUS	P	PD	VUS	P	P	P
RPE65	NM_000329.3	c.119G > A	28.1	VUS	P	P	VUS	P	PD	P	P	LP	LP
ABCA4	NM_000350.3	c.91 T > C	27.7	VUS	B	P	VUS	P	PD	P	P	LP	LP
ABCA4	NM_000350.3	c.214G > A	27.6	P	VUS	P	VUS	n.a	PD	P	P	P	P/LP
CNGA3	NM_001298.3	c.991G > C	27.3	P	P	P	VUS	P	PD	P	P	LP	n.a
RDH5	NM_002905.5	c.758 T > G	27.2	B	P	VUS	VUS	VUS	PD	VUS	B	VUS	n.a
RDH12	NM_152443.3	c.609C > A	27.1	VUS	P	P	VUS	P	PD	P	P	P	P
CLRN1	NM_174878.3	c.461 T > G	27	B	P	VUS	VUS	VUS	PD	P	VUS	VUS	LP
CDH23	NM_022124.6	c.7198C > T	27	P	VUS	P	VUS	VUS	PD	P	P	VUS	n.a
ABCA4	NM_000350.3	c.2023G > A	26.9	VUS	P	P	VUS	n.a	PD	B	P	VUS	CFL
CEP290	NM_025114.4	c.148C > T	26.7	VUS	VUS	B	VUS	B	PD	B	VUS	VUS	LP
CNGA3	NM_001298.3	c.955 T > C	26.6	VUS	P	P	VUS	P	PD	P	P	P	P/LP
CERKL	NM_201548.5	c.316C > A	26.5	VUS	VUS	VUS	VUS	P	PD	VUS	B	VUS	CFL
CNGA3	NM_001298.3	c.952G > A	26.5	VUS	P	P	VUS	P	PD	VUS	VUS	VUS	n.a
CNGA3	NM_001298.3	c.847C > T	26.5	VUS	P	P	VUS	P	PD	P	P	P	P/LP
TULP1	NM_003322.6	c.1274 T > C	26.4	VUS	VUS	P	VUS	P	PD	P	P	LP	n.a
SEMA4A	NM_022367.4	c.1049 T > G	26.1	VUS	VUS	P	VUS	P	PD	P	P	LP	P
GUCY2D	NM_000180.4	c.917A > T	26	VUS	VUS	VUS	VUS	P	PD	P	P	VUS	n.a
BBS12	NM_152618.3	c.1589 T > C	26	VUS	VUS	VUS	VUS	P	PD	P	P	VUS	n.a
TMEM67	NM_153704.6	c.1127A > C	26	VUS	VUS	VUS	VUS	VUS	PD	VUS	VUS	P	P
ARL6/BBS3	NM_001278293.3	c.281 T > C	25.8	VUS	P	P	VUS	P	n.a	VUS	P	LP	P
DHX38	NM_014003.4	c.995G > A	25.8	VUS	VUS	VUS	VUS	B	PD	P	B	VUS	VUS
EYS	NM_001142800.2	c.7187G > C	25.7	VUS	VUS	P	B	P	PD	VUS	P	VUS	VUS
ASRGL1	NM_001083926.2	c.532G > A	25.6	P	P	P	VUS	P	PD	P	P	LP	n.a
PDE6B	NM_000283.4	c.1160C > T	25.6	VUS	P	P	VUS	P	PD	P	P	LP	P
IMPDH1	NM_000883.4	c.931G > A	25.6	VUS	P	n.a	VUS	P	PD	P	VUS	P	P
CRB1	NM_201253.3	c.3101G > C	25.5	VUS	n.a	P	VUS	VUS	n.a	P	P	LP	n.a
CRB1	NM_201253.3	c.3962G > C	25.5	P	n.a	P	VUS	P	PD	P	P	P	n.a
RDH12	NM_152443.3	c.619A > G	25.5	VUS	P	P	VUS	P	PD	P	VUS	LP	P
BBS12	NM_152618.3	c.1616G > T	25.4	VUS	VUS	VUS	VUS	P	PD	P	VUS	LP	VUS
RHO	NM_000539.3	c.448G > A	25.3	P	VUS	VUS	VUS	P	PD	B	VUS	P	LP
LCA5	NM_001122769.3	c.652C > G	25.3	VUS	VUS	VUS	B	B	PD	P	P	VUS	n.a
TULP1	NM_003322.6	c.1138A > G	25.2	VUS	P	VUS	VUS	P	B	P	VUS	VUS	n.a
ABCA4	NM_000350.3	c.5243G > A	25.1	VUS	B	VUS	B	VUS	PD	VUS	B	VUS	P
RPGRIP1	NM_020366.4	c.2480G > T	25.1	VUS	VUS	VUS	VUS	P	PD	P	VUS	VUS	P
MKKS	NM_170784.3	c.280 T > C	25	VUS	VUS	P	VUS	P	PD	VUS	B	VUS	n.a
CNGA3	NM_001298.3	c.827A > G	24.8	VUS	P	P	VUS	P	PD	P	P	LP	LP
GUCY2D	NM_000180.4	c.2189 T > C	24.8	VUS	VUS	P	B	P	PD	P	P	LP	n.a
RPGRIP1	NM_020366.4	c.2656C > T	24.7	VUS	VUS	P	VUS	B	PD	VUS	VUS	VUS	n.a
CRB1	NM_201253.3	c.433 T > C	24.6	VUS	n.a	P	VUS	P	PD	P	P	LP	n.a
RDH5	NM_002905.5	c.536A > G	24.5	VUS	P	P	VUS	P	PD	VUS	VUS	LP	P
SEMA4A	NM_022367.4	c.1033G > C	24.4	VUS	VUS	P	VUS	P	PD	VUS	VUS	LP	VUS
PDE6B	NM_001145292.2	c.938C > T	24.3	VUS	P	B	VUS	VUS	B	B	B	VUS	LP
CRB1	NM_201253.3	c.3296C > A	24.3	VUS	n.a	VUS	VUS	VUS	PD	VUS	VUS	VUS	n.a
PRCD	NM_001077620.3	c.2 T > C	24.2	VUS	VUS	n.a	VUS	P	n.a	B	VUS	P	P
BBS5	NM_152384.3	c.2 T > A	24.2	VUS	VUS	n.a	VUS	P	n.a	B	VUS	P	P
CNGA3	NM_001298.3	c.822G > T	24.1	VUS	P	P	VUS	P	PD	P	P	P	CFL
CNGA3	NM_001298.3	c.1306C > T	24	VUS	P	P	VUS	n.a	PD	P	P	P	P
CLRN1	NM_174878.3	c.92C > T	23.9	VUS	VUS	B	VUS	VUS	PD	VUS	VUS	LP	P
BBS2	NM_031885.5	c.443A > T	23.8	VUS	VUS	n.a	B	P	PD	VUS	P	VUS	n.a
ZNF513	NM_144631.6	c.1015 T > C	23.8	VUS	VUS	B	VUS	VUS	PD	P	VUS	B	VUS
GRM6	NM_000843.4	c.824G > A	23.7	VUS	n.a	VUS	VUS	VUS	PD	VUS	B	VUS	VUS
PDE6A	NM_000440.3	c.304C > A	23.6	VUS	P	VUS	B	n.a	PD	VUS	B	P	CFL
IMPDH1	NM_000883.4	c.676G > A	23.6	VUS	P	n.a	VUS	P	B	P	B	VUS	n.a
RDH5	NM_002905.5	c.602C > T	23.6	VUS	VUS	P	B	VUS	PD	VUS	VUS	VUS	n.a
NMNAT1	NM_022787.4	c.25G > A	23.6	VUS	B	P	B	VUS	PD	B	P	P	P
CLCC1	NM_001377458.1	c.75C > A	23.6	VUS	P	P	VUS	P	PD	VUS	P	VUS	P
EYS	NM_001142800.2	c.8299A > T	23.4	VUS	B	VUS	B	VUS	PD	B	B	LB	n.a
TULP1	NM_003322.6	c.1307C > G	23.4	VUS	VUS	B	VUS	P	B	P	VUS	VUS	n.a
AIPL1	NM_014336.5	c.116C > A	23.4	B	P	P	VUS	P	PD	P	P	VUS	n.a
CRB1	NM_201253.3	c.2234C > T	23.1	VUS	n.a	P	VUS	n.a	PD	P	P	P	P/LP
BEST1	NM_004183.4	c.418C > G	22.8	VUS	B	P	VUS	P	PD	B	VUS	LP	CFL
CRB1	NM_201253.3	c.2536G > A	22.8	VUS	n.a	P	VUS	P	PD	P	P	P	P
BBS1	NM_024649.5	c.442G > A	22.7	VUS	n.a	VUS	VUS	n.a	PD	VUS	B	VUS	CFL
GUCY2D	NM_000180.4	c.530G > C	22.7	VUS	B	VUS	VUS	P	PD	VUS	VUS	VUS	n.a
RPE65	NM_000329.3	c.1087C > A	22.7	VUS	VUS	VUS	VUS	P	B	B	B	P	P
NMNAT1	NM_022787.4	c.547C > T	22.7	VUS	B	P	B	VUS	PD	VUS	VUS	VUS	VUS
CRB1	NM_201253.3	c.1459 T > C	22.6	VUS	n.a	VUS	VUS	VUS	PD	P	VUS	VUS	B/LB
IFT43	NM_001102564.3	c.100G > A	22.6	VUS	B	B	B	n.a	PD	VUS	VUS	VUS	CFL
RPGRIP1	NM_020366.4	c.1639G > T	22.5	VUS	B	B	B	n.a	PD	B	B	B	B/LB
GRM6	NM_000843.4	c.2267G > A	22.3	VUS	n.a	VUS	B	P	B	VUS	B	VUS	P
CRB1	NM_201253.3	c.2966 T > C	22.2	VUS	n.a	VUS	VUS	P	PD	VUS	VUS	VUS	n.a
RDH5	NM_002905.5	c.668A > C	22.1	VUS	B	VUS	B	VUS	PD	VUS	B	VUS	n.a
SEMA4A	NM_022367.4	c.2138G > A	21.3	VUS	B	B	B	n.a	B	B	B	B	B/LB
FAM161A	NM_001201543.2	c.1139G > T	18.53	VUS	B	VUS	VUS	P	PD	P	VUS	VUS	VUS
ALMS1	NM_001378454.1	c.5242A > G	17.8	VUS	n.a	n.a	VUS	n.a	n.a	n.a	n.a	B	LB
NR2E3	NM_014249.4	c.227G > A	16.07	B	B	B	n.a	n.a	PD	n.a	n.a	LP	CFL
AIPL1	NM_014336.5	c.773G > C	15.35	B	B	B	VUS	VUS	PD	VUS	B	VUS	LP
CRB1	NM_201253.3	c.3347 T > C	9.398	B	n.a	VUS	VUS	VUS	B	B	B	VUS	n.a
RP1	NM_006269.2	c.1118C > T	9.092	B	B	VUS	B	n.a	B	B	VUS	B	B/LB
RAX2	NM_001319074.4	c.374G > A	1.497	B	B	B	VUS	B	n.a	n.a	n.a	LB	n.a
RP1	NM_006269.2	c.2005G > A	1.173	B	B	B	VUS	n.a	B	B	B	VUS	VUS
GUCY2D	NM_000180.4	c.2384G > A	32	P	VUS	P	B	n.a	PD	VUS	P	P	VUS

*MP* MutPred, *MT* Mutation taster, *MA* Mutation assessor, *LRT* Likelihood ratio test, *PP-2* PolyPhen-2, *PRO* PROVEAN, *SIFT* Sorting intolerant from tolerant, *ACMG* The American college of medical genetics and genomics, *B* Benign, *LB* Likely benign, *B/LB* Benign/Likely benign, *P* Pathogenic, *LP* Likely pathogenic, *P/LP* Pathogenic/Likely pathogenic, *PD* Probably/Possibly damaging, *VUS* Variant of uncertain significance, *CFL* Conflicting, *NA* Not available

In summary, eight alleles were eligible to be classified as benign/likely benign consistently by the ACMG standards, ClinVar classification system, and by majority of the online *in-silico* predictors. These eight benign/likely benign missense alleles are further detailed in Table 3, and include *SEMA4A* (p.Arg713Gln) [72], *USH2A* (p.Ser2445Phe) [73], *RPGRIP1* (p.Ala547Ser) [74], *RP1* (p.Thr373Ile) [75], *ZNF513* (p.Cys339Arg) [7], *ALMS1* (p.Lys1748Glu) [76], *RAX2* (p.Gly125Glu) [77], and *EYS* (p.Thr2777Ser) [78].

**Table 3:** List of missense variants classified as ‘benign or Likely benign’ by the ACMG guidelines

	**cDNA change**	**Protein change**	**gnomAD MAF**	**Disease**	**Population**	**Consanguinity**	**Zygosity**	**ACMG**
*SEMA4A*	c.2138G > A	p.(Arg713Gln)	0.03651	adRP	N.D	No	Heterozygous	Benign
*USH2A*	c.7334C > T	p.(Ser2445Phe)	0.000841	arUSH2	KPK	No	Homozygous	Benign
*RPGRIP1*	c.1639G > T	p.(Ala547Ser)	0.2038	arCRD	N.D	Yes	Homozygous	Benign
*RP1*	c.1118C > T	p.(Thr373Ile)	0.01215	arRP	N.D	Yes	Homozygous	Benign
*ZNF513*	c.1015 T > C	p.(Cys339Arg)	0.0001668	arRP	Punjab	Yes	Homozygous	Benign
*ALMS1*	c.5242A > G	p.(Lys1748Glu)	NA	arAS	Punjab	Yes	Homozygous	Benign
*RAX2*	c.374G > A	p.(Gly125Glu)	NA	arRD	Punjab	Yes	Homozygous	Likely Benign
*RAX2*	c.8299A > T	p.(Thr2777Ser)	NA	arRP	Punjab	Yes	Homozygous	Likely Benign

## Discussion

This study provides an overview of the existing clinical and genetic aspects of IRDs in Pakistani families based on published reports. Majority of the IRDs families in the published reports belonged to two major ethnic Pakistani populations i.e. Punjab and Khyber Pakhtunkhwa. Other ethnic Pakistani populations such as Sindh, Baluchistan and Gilgit-Baltistan were only marginally represented in the available medical literature. Consistent with the traditional practice of endogamy in the country [79–81], over 70% IRDs cases in this study were found among children whose parents were consanguineously married. Consequently, recessively inherited IRDs were disproportionately high (> 95%) in the current report as opposed to dominant cases. Our findings reiterate the fact that consanguinity-driven homozygosity mapping can greatly leverage identification of novel disease genes in recessively inherited Mendelian disorders in endogamous populations as previously shown [82, 83]. For example, at least 12 IRDs-associated genes have been first identified/reported in Pakistani families [84, 85].

Of the total retrieved variants detected in IRDs families of Pakistani origin, ~ 80% were considered as ‘rare’ since they were reported only once from the Pakistani population. The remainder ~ 20% variants were observed at least twice in Pakistani families, hence they were considered as ‘recurrent’ alleles. Of the recurrent alleles, a frameshift mutation in *LCA5* gene (NM_001122779.3:c.1151delC;p.Pro384GlnfsTer18) was independently reported seven times [76, 86–91], and thus considered to be the topmost commonly reported allele in Pakistan.

While ethnic affiliations of index cases carrying c.1151delC allele were not provided in five out of seven studies, c.1151delC allele was independently reported in two unrelated families from KPK and Punjab provinces. The second most recurrent allele was a missense substitution (c.1138A > G:p.Thr380Ala) in the *TULP1* (NM_003322.6) gene which was reported by five independent studies. Of them, two families belonged to ethnically matched KPK population, and thus possibly related. However, one family carrying c.1138A > G allele belonged to Punjab province. No data on the ethnic affiliation was available in the remaining two reports [88–90, 92, 93]. In addition to the likelihood that these alleles constitute hotspot mutations, frequent occurrence of these recurrent alleles in multiple and ethnically matched Pakistani families might indicate a founder effect in the society.

Our findings, of RP as the most leading IRD type in Pakistani families (41%), align with several previous studies suggesting RP as the most frequently reported form of IRDs in world populations [94–98]. Also, we have found that RP is the most genetically heterogeneous disorder among IRDs in Pakistani population as mutations in 37 different RP-associated genes were identified. Nonetheless, Pakistani families somehow present unique genetic architecture of IRDs. For example, unlike *ABCA4* and/or *USH2A* gene mutations which are considered as a major etiology of the IRDs cases worldwide [96–98], we have found that *PDE6A* gene mutations are the leading cause of IRDs in Pakistani families. While disease-causing mutations in *SNRNP200* gene are known to cause adRP [99–101], *SNRNP200* gene mutation correlated with arRP in a Pakistani IRD family [102]. Lastly, detection of rare forms of IRDs notably *STX3*-associated intestinal-retinal syndrome [103], *VPS13B-*associated Cohen syndrome [104], and *SLC6A6*-associated taurine transporter (TauT) deficiency disorders [105] in Pakistani families not only points towards the distinctive genetic nature of this population but it also highlights its potential in medical research.

Our CADD score analysis of all missense variants that are described in this report revealed two alleles with lowest CADD-PHRED scores. These included *RP1* (c.2005G > A; p.Ala669Thr), and *RAX2 (*c.374G > A; p.Gly125Glu) with a CADD-PHRED scores of 1.173 and 1.497, respectively. Though our *in-silico* findings about these alleles are not final, we recommend further researcher to further investigate these two missense variants in order to fully characterize their impact on protein structure and/or function.

## Conclusions

This study provides a comprehensive overview of IRDs in Pakistani families over a period of 25 years (1999–2023). Our analysis reaffirms the fact that majority of the prevalent IRDs cases in Pakistan are recessively inherited, and that they mostly appeared due to the bi-allelic inheritance of rare pathogenic mutations from both parents. Undoubtedly, RP was the most frequently occurring IRD in Pakistan followed by LCA. Overall, *PDE6A* gene mutations was the leading cause of IRDs in Pakistani families followed by mutations in *TULP1* gene. Altogether, marked genetic and allelic and heterogeneity was observed in the Pakistani IRDs families. In summary, Pakistani families are notable in expressing both common and rare Mendelian disorders such as Cohen syndrome, intestinal-retinal syndrome, and taurine transporter deficiency possibly due to the traditional practice of endogamy in the society.

### Limitations

Since the data presented in this study were all retrieved from published reports, and further validated/curated using online databases, we foresee certain limitations in our study. For example, we do not claim causality of variants (if any) presented in this report as it does not fall under the purview of our study. Despite our own efforts, we do see the possibility of overlooking certain relevant literature on the subject. Incomplete information, inconsistencies or in some cases errors seen in the retrieved data may have skewed our own analysis. It is pertinent to mention here that all calculations about genomic variants were based on the total number of alleles (*n* = 277) reported in the 126 research articles. We were unfortunately unable to assess objectively the total number of families and/or patients affected by these 277 alleles due to the inadequate information provided in the literature. Nevertheless, published reports emerged mostly from two provinces of Pakistan i.e. Punjab and Khyber Pakhtunkhwa. Therefore, we recommend taking care while extrapolating our findings to other ethnic Pakistani populations like Sindh, Baluchistan and Gilgit-Baltistan.

### Supplementary Information

**Additional file 1. **

**Additional file 2. **

**Additional file 3. **

## Data Availability

All data generated or analyzed during this study are included in this published article [and its supplementary information files].

## Abbreviations

ACMG: The American College of Medical Genetics and Genomics
adRP: Autosomal Dominant Retinitis Pigmentosa
arRP: Autosomal Recessive Retinitis Pigmentosa
AS: Alström syndrome
BBS: Bardet-Biedl syndrome
CADD: Combined Annotation Dependent Depletion
CD/CRD: Cone dystrophy or Cone rod dystrophy
CHM: Choroideremia
COH: Cohen syndrome
CSNB: Congenital Stationary Night Blindness
EORD: Early onset retinal dystrophy
FA: Fundus Albipunctatus
gnomAD: Genome aggregation database
HGMD: Human Gene Mutation Database
HJMD: Hypotrichosis juvenile macular dystrophy
IRDs: Inherited retinal degenerations
JBTS: Joubert syndrome
LCA: Leber’s congenital amaurosis
MD: Macular degeneration
MKS: Meckel Syndrome
RDMVID: Retinal dystrophy with microvillus inclusion disease
RP: Retinitis pigmentosa
RPA: Retinitis punctata albescens
RS: Retinoschisis
SIFT: Sorting Intolerant from Tolerant
SLS: Senior-Loken syndrome
STGD: Stargardt disease
USH: Usher Syndrome, and
ZS: Zellweger syndrome
